# Versatility of the subscapular system of flaps in head and neck oncologic reconstruction

**DOI:** 10.1097/MOO.0000000000000771

**Published:** 2021-10-19

**Authors:** Alberto Deganello, Vittorio Rampinelli, Tommaso Gualtieri, Cesare Piazza

**Affiliations:** a Unit of Otorhinolaryngology – Head and Neck Surgery, ASST Spedali Civili of Brescia, Brescia, Italy; b Department of Medical and Surgical Specialties, Radiological Sciences, and Public Health, University of Brescia, School of Medicine, Brescia, Italy

**Keywords:** chimeric free flap, latissimus dorsi flap, maxillary reconstruction, oromandibular reconstruction, parascapular free flap, scapular tip free flap

## Abstract

This review analyzes the different ways of applying the subscapular system of flaps (SSSF) as a convenient and versatile solution for a large variety of head and neck oncologic defects.

**Recent findings:**

The ventral approach permits safe and efficient harvest of various chimeric SSSF in a supine position, thus allowing simultaneous flap preparation and tumor ablation. Conformational studies have revealed how similar the tip of the scapula is to the hard palate in terms of dimensions, shape, and conformation. This has led to favor horizontal placement of the scapular tip for palate reconstruction in most instances, addressing the vertical extension of the postmaxillectomy defect using denuded bony grafts surrounded by well vascularized chimeric muscular components.

**Summary:**

The SSSF possesses an unparalleled versatility to efficiently address small-medium sized soft tissue defects up to vast and complex composite resections. The chimeric components of these flaps benefit from a considerable independency provided by the length of the named arteries arising from the thoracodorsal pedicle, offering a high degree of freedom to accomplish the required in-setting. This reconstructive option should be implemented in every head and neck surgical team and offered to suitable patients.

## INTRODUCTION

Surgical resection of head and neck tumors may produce a wide variety of soft and bone tissues losses and, therefore, in order to accomplish effective restoration of their original form and function, every head and neck defect should be precisely anticipated and evaluated in terms of lack of support, cover, and lining. In this light, the subscapular system of flaps (SSSF) has been recognized as one of the most versatile for head and neck oncologic reconstructions [1–2], since it can provide multiple combinations of independent components, potentially harvested in a single chimeric flap.

A comprehensive detailed description of the subscapular vascular anatomy and its possible variants is beyond the scope of this work and was summarized in a recent review [3]. The subscapular artery divides into two main branches, the circumflex artery and the thoracodorsal artery (TDA). The circumflex artery provides vascularization to two large parascapular fasciocutaneous flaps and to the lateral portion of the scapular bone. When the harvest includes the primary bony branches from the circumflex artery to the lateral scapular border, the resulting pedicle, formed by the circumflex scapular vessels or, more cranially, by the subscapular artery and vein, is quite short, not exceeding 3–4 cm. Therefore, the inclusion of the tip of the scapula as a source of vascularized bone through the angular branch of the TDA has gained increasing favor since 2010 [4,5], given the advantage of relying on a long pedicle and the possibility to effectively provide adequate blood supply for a relevant portion of the inferior scapula and adjacent muscles. In fact, bony segments up to 12 cm in length have been successfully applied in association with muscle cuffs taken from the teres major and serratus anterior [6^▪^].

The TDA usually divides into three branches: the first in descending order is represented by a branch for the latissimus dorsi and overlying skin. As recently described [7], branches from the main thoracodorsal pedicle can travel directly to the infra-axillary skin without piercing muscles, making possible septocutaneous harvest of the TDA perforator (TDAP) flap. The second branch is mostly represented by a vascular pedicle for the serratus muscle (with the possibility of including a segment of rib) and overlying skin, whereas the third is the angular branch for the tip of the scapula and teres major muscle. The tip of the scapula is a thin, triangular shaped bone presenting obvious tridimensional limitations when intended to replace the maxilla (a cuboidal structure) [8] or the mandible (a long bony arc with straight and curved portions). Hence, various in-setting strategies have been proposed to replace such composite defects, and no standardized and universally accepted policy exists.

This review analyzes how the SSSF plays an important role in a variety of head and neck oncologic reconstructions, from simple medium-sized soft tissue replacements to the restoration of complex three-dimensional, composite defects. Our strategies of using various combinations of chimeric harvests for bony and composite reconstructions will be also outlined. 

**Box 1:**
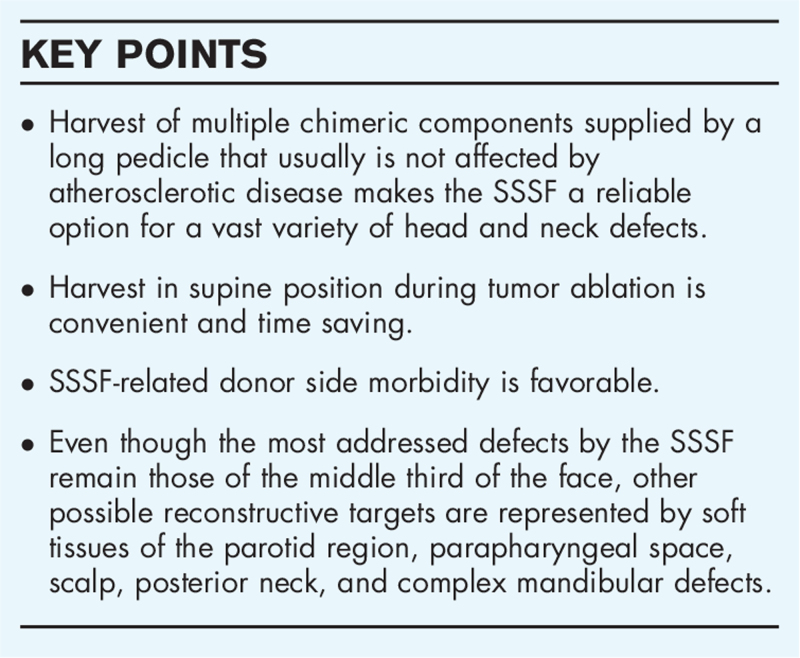
no caption available

## HARVESTING POSITION

One of the most discouraging aspects concerning the SSSF has been traditionally the claim that when using such free flaps the patient should be turned in a lateral decubitus, or even in a prone position, and then turned back, with obvious increase of time spent, surgical complexity, and overall workload for the operatory room team. Patient positioning at 30° lateral decubitus was described to overcome the need of turning the patient, allowing a two-team approach [5]; however, in agreement with a series of 53 consecutive flaps by Ferri *et al.*[9^▪▪^], it is our experience that every type of SSSF harvest, from the septocutaneous TDAP to the more complicated composite chimeric flaps, can be easily and safely raised in a supine position during simultaneous tumor resection.

Using such an approach, the patient is placed with the thorax projecting a few centimeters over the lateral edge of the surgical bed at the selected side, while a latex wedge is located under the ipsilateral iliac crest. With one hand the assistant holds the patient's forearm, while with the other the shoulder is sustained during the entire harvest. The arm is abducted less than 90° with the forearm flexed at 90°. The reconstructive assistant is standing next and immediately behind the first assistant of the ablative team. During tumor resection (including neck dissection, maxillectomy, parotidectomy, oral cavity resection, etc.), the flap is simultaneously harvested from the contralateral side. The skin incision starts from the axillary midpoint, following down the projection of the anterior edge of the latissimus dorsi muscle, and is adjusted for the intended skin paddle. The anterior edge of the latissimus dorsi is first identified, while ventral dissection of the muscle reveals its muscular hilum and the entire circumflex and thoracodorsal vascular anatomy, allowing the harvesting of the entire gamma of SSSF needed in this area. With such a ventral approach, after identification of the angular branch of the TDA, if a tip scapula free flap is planned, it is very convenient to pinch the tip of the bone with a solid Backhaus clamp, since its anterior and lateral traction greatly facilitates dissection of the flap and enhances visualization before osteotomies.

## MEDIUM AND LARGE SIZED SOFT TISSUE DEFECTS

**FIGURE 1:**
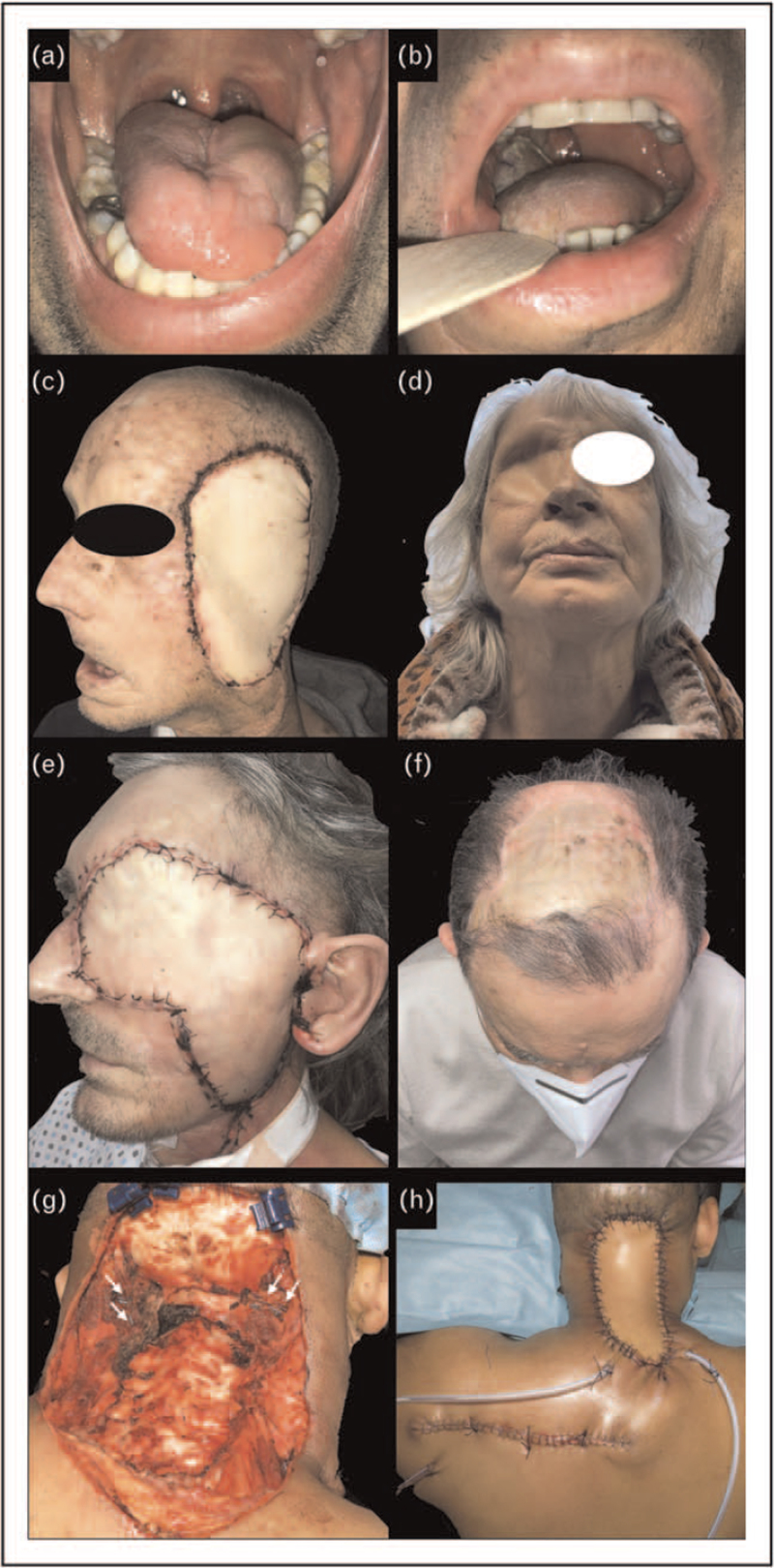
Soft tissue reconstructions. (a) tongue reconstruction with septocutaneous TDAP. (b) tongue and lateral oropharyngeal wall reconstruction following total glossectomy with free latissimus dorsi myocutaneous flap. (c) transposition of a pedicled latissimus dorsi myocutaneous flap following total auriculectomy, radical parotidectomy with large skin resection for a neglected cutaneous squamous cell carcinoma. (d) orbital reconstruction with a myocutaneous latissimus dorsi free flap after endoscopic craniectomy with orbital exenteration. (e) transposition of a free latissimus dorsi myocutaneous flap following orbito-zygomatic removal with dural resection, radical parotidectomy, segmental mandibular resection, for a neglected squamous cell carcinoma of the eyelids. (f) scalp reconstruction after large subperiosteal resection of a large cutaneous squamous cell carcinoma of the vertex, using the serratus anterior muscle free flap with a split thickness skin graft. (g,h) posterior scalp and neck soft tissues reconstruction after removal of an extensive paravertebral myxoid sarcoma recurrent after inappropriate surgery and radiotherapy. A left, horizontally oriented parascapular fascio-cutaneous free flap was harvested and revascularized on the ipsilateral occipital artery and vein. White arrows indicate left and right occipital arteries and veins.

Soft tissue defects within the neck, oral cavity, oropharynx, hypopharynx and larynx may require transposition of pliable tissues, restoring the separation between the aerodigestive tract and neck compartments, with the aim of enhancing the residual functions of preserved structures around the resection, especially when the tongue is involved. For these purposes, fascio-cutaneous flaps such as the radial forearm or the anterolateral thigh are extensively used. However, the SSSF can offer competing alternatives, represented by the parascapular fascio-cutaneous, the TDAP (Fig. 1a) and, for larger defects, the latissimus dorsi and the serratus anterior myo-cutaneous flaps.

In case of total glossectomy defects, especially if the larynx has been preserved, the myo-cutaneous latissimus dorsi free flap offers an adequate amount of bulk to create a new tongue with vertical projection thus providing functional tongue-palate competence and allowing to squeeze the bolus from the oral cavity toward the oropharynx (Fig. 1b). This free flap favorably compares with those harvested from the abdomen in terms of donor side cosmesis and morbidity, without the risk of laparocele formation as frequently observed in elderly or obese subjects in spite of any attempt of abdominal wall reconstruction. Moreover, reinnervation of the latissimus muscle through one hypoglossal nerve, even though not able to recreate any type of neotongue movement, may greatly maintain its tone and volume, thus reducing the progressive atrophy of the reconstruction itself. For total glossectomy defects, a chimeric transposition of TDAP and scapula tip flap has been recently proposed [10]. The tip of the scapula is fixed to the inferior border of the mandible, with the aim of keeping overtime a stable bony support and prevent a downwards displacement of the TDAP reconstructing the neotongue in a dome-like shape.

In case of pharyngo-laryngectomy or total glosso-laryngectomy defects, the latissimus dorsi myo-cutaneous flap represents a reliable reconstructive option that can be transposed as either a free or pedicled flap. However, these techniques should be considered as second-line options, especially for muscular and thick males, in which the myo-cutaneous paddle of latissimus dorsi may represent an obstacle in distal suturing to the cervico-mediastinal stump of the esophagus, thus increasing the risk for pharyngo-cutaneous fistula and stricture. Therefore, in these clinical scenarios, thinner fascio-cutaneous free flaps such as radial forearm and anterolateral thigh still represent the workhorse donor sites [11–13].

On the contrary, the transposition of a pedicled latissimus dorsi flap is a reliable solution for a wide spectrum of large head and neck mucosal and skin defects (Fig. 1c). Its wide arc of rotation permits this pedicled flap to virtually reach as high as the vertex. In this occasion, the flap is carefully passed over or through the pectoralis major muscle, making it very useful in salvage cases of vessel depleted necks or when the pectoralis major flap has been previously transposed.

To fill the gap after extended parotid resections, the TDAP flap can be de-epithelized to replace the tissue loss resulting from extirpation of the parotid gland and, when the overlying skin is involved by tumor, this flap may be transposed as a fascio-cutaneous paddle to restore the cutaneous defect [14]. The SSSF is also particularly useful in case of total parotidectomy defects associated with parapharyngeal space clearance, especially when including mucosal resection of the lateral wall of the nasopharynx and/or oropharynx. In these cases, a chimeric flap of latissimus dorsi and serratus muscles, with one component (usually the serratus anterior) filling the parapharyngeal space and sutured around the mucosal defect, and the other muscle placed over the facial nerve, enables achieving good facial contour with simultaneous optimal pharyngeal closure and parapharyngeal space obliteration.

For neck, facial, and scalp reconstructions, whenever a large amount of pliable tissue is needed, and dural exposure or transgression with ensuing reconstruction has been accomplished, the SSSF provides excellent solutions (Fig. 1d-f). It undoubtedly represents the first-choice option in case of posterior neck/occipital skin and soft tissues defects. The prone position of the surgical resection, in fact, allows a direct and easy approach to the SSSF of both sides, with the possibility of revascularizing the flap harvested through the occipital artery and vein (Fig. 1g,h). Large resections of the scalp with removal of the pericranium and exposure of cranial bones, or in combination to alloplastic cranioplasty reconstruction, pose particularly challenging reconstructive dilemmas. The latissimus dorsi or serratus anterior muscular flaps, with a split thickness skin graft on the top, provide optimal one stage solutions [15] (Fig. 1f).

## MAXILLARY AND ORBITO-ZYGOMATIC DEFECTS

In the last decade, the SSSF has gained increasing popularity until becoming the preferred method for reconstruction of postmaxillectomy defects [1,5,9^▪▪^,^16^]. The complex tridimensional requirements needed to provide adequate rehabilitation after this kind of surgery pose a serious challenge to the reconstructive surgeon. In fact, to date, no standardized reconstructive strategy has been established, and various in-setting modalities have been proposed and implemented.

**FIGURE 2:**
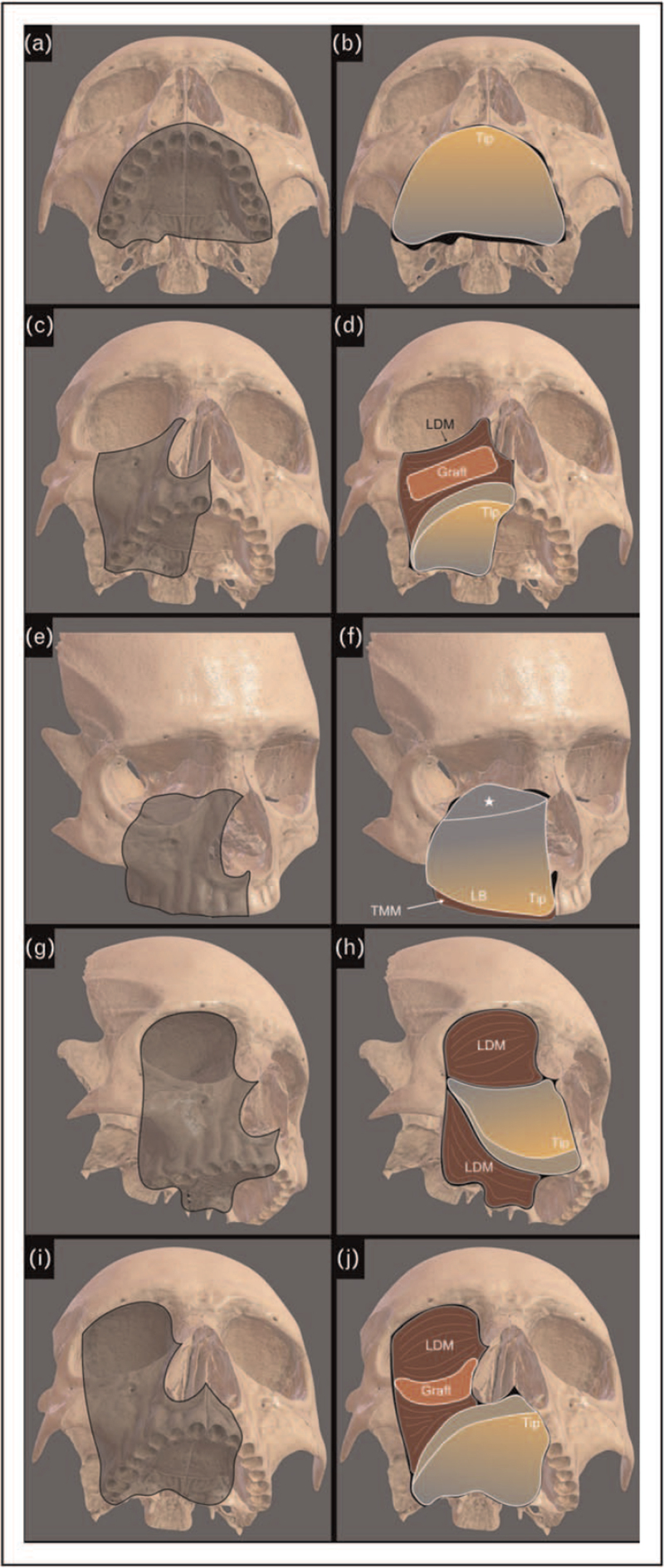
Diagram of maxillary reconstructions. (a-b) horizontal placement of the scapula tip flap for total palate reconstruction. (c-d) horizontal placement of the scapula tip flap for total palate reconstruction, with the latissimus dorsi chimeric component (LDM) obliterating the space between the scapula and the orbit and wrapping the bone graft replacing the anterior maxillary wall. (e-f) vertical placement of the scapula with the tip of scapula lateral border replacing the alveolar ridge and the teres major muscle (TMM) reflected intraorally to ensure palate closure. The superior portion of the bone is ‘greenstick’ fractured to reconstruct the resected orbital floor (white star). (g-h) vertical placement of the scapula with the tip replacing the most anterior portion of the alveolar ridge, the lateral border replacing the resected alveolar ridge, the osteotomy line replacing the inferior orbital rim, and the chimeric latissimus dorsi (LDM) obliterating the orbito-maxillary defect and reconstructing the hard palate between soft palate and bony reconstruction. (i-j) horizontal placement of the scapula tip flap for subtotal palate reconstruction, with the latissimus dorsi chimeric component (LDM) obliterating the orbito-maxillary cavity and wrapping a bone graft to replace the inferior orbital rim and anterior maxillary wall. The skull representations at the base of the diagrams are from Head Atlas App, UpSurgeOn Srl, Via Cascina Venina, 7, 20090 Assago MI.

The bony component of the scapular tip flap provides a triangular flat bone, whereas maxillectomy defects greatly differ in terms of tridimensional bony loss, and thus the reconstructive procedure must overcome and minimize this discrepancy by adequate customization of the reconstruction itself. Taking advantage from the evidence that conformational studies have revealed how similar the tip of the scapula is to the hard palate in terms of dimensions, shape, and conformation [17], in our practice the anticipated defect is evaluated in terms of both amount of palate resection and its vertical height. The osseous reconstruction of the anterior and anterolateral alveolar ridge represents the first crucial step, since it is essential for a correct middle-third facial projection, and to sustain additional reconstructive/prosthetic components. Next, depending on the vertical extension of the resection, the anterior maxillary wall, orbital rim and floor, and orbital contents are also addressed. In this light, any hard palate defect with anterior involvement beyond one canine (Okay class II defects) and defects with removal of 50% or more of the superior alveolar ridge (Okay class III, Brown class B and C) [18,19] are reconstructed by placing the scapular tip free flap in a horizontal position. The harvest of the contralateral scapula provides an ipsilateral vascular pedicle with the lateral scapular border reconstructing the most involved alveolar side, the scapular angle replacing the most anterior portion of the superior alveolar ridge, and the concave surface of the bony flap facing the oral cavity, as the new vault of the hard palate (Fig. 2a,b).

Proportionally to the vertical extension of the defect, bony grafts from redundant portions of the scapular flap, obtained by setting the osteotomy more cranially than required, are used to replace the anterior maxillary wall (Brown class 2) or to reconstruct the orbital floor (Brown class 3, Okay subclass f). These grafts must be completely denuded from their original muscular attachments, and must be surrounded by well vascularized tissue (Fig. 2c,d). When replacing the orbital floor, the graft is fixed by securing correct alignment of the eyes, and is sustained, covered and nourished by the chimeric latissimus dorsi muscle that obliterates the dead space between the scapular tip flap and the orbit. The anterior wall of the maxilla can also be replaced using a bony graft wrapped by the latissimus dorsi muscle. All grafts are fixed to the surrounding bone with miniplates or with nonabsorbable sutures that must be adequately covered by muscle and placed as faraway as possible from the facial skin incisions to avoid late exposures. Another possible way of dealing with a total maxillectomy defect with loss of orbital floor support has been originally described by Clark and coworkers [20], and subsequently applied by others [8,16]. It is based on using the contralateral tip of scapula vertically, positioning the tip antero-medially in correspondence with the anterior nasal spine, and fixing it to the opposite residual alveolar ridge. The dorsal (convex) part of the bone is directed forward and subsequently covered by facial skin. The lateral border of the bone flap is used to reconstruct the resected alveolar ridge, while teres major muscle fills the gap of the residual hard palate resection. The superior and lateral portion of the scapular bone is fixed to the zygomatic bone and part of it is ‘greenstick’ fractured to sustain the eyeball. This part of the scapula, usually thin and peripheral, should maintain adequate vascularization through the dorsal periosteum of the bone and adjacent muscles. A micro- or miniplate must be usually added to fix it in the right position and avoid displacement with eyeball misalignment and consequent diplopia (Fig. 2e,f). As an alternative, the orbital floor can also be reconstructed using preplated titanium meshes [21]. However, the risk of subsequent exposure and extrusion after radiotherapic adjuvant treatment must be carefully balanced.

When maxillectomy includes orbital clearance (Brown class 4), the in-setting of the scapula tip depends upon the amount of hard palate resection. If the contralateral alveolar ridge is preserved (Okay class IIf, Brown class A4), the scapular tip is placed vertically, with the angle replacing the most anterior and medial portion of the resected superior alveolar ridge. The medial scapular border is fixed against the nasal osteotomy, the lateral scapular border replaces the resected alveolar ridge and is fixed to the malar bone, and the osteotomy edge replaces the resected orbital rim and is shaped/drilled to match the contralateral healthy side. The chimeric latissimus dorsi muscular portion is used to obliterate the orbital-maxillary cavity, and to reconstruct the remaining posterior portion of the hard palate [22] (Fig. 2g,h).

If the hard palate resection includes more than 50% of the alveolar ridge (Okay class IIIf, Brown class B4 and C4), the scapular tip is fixed horizontally thus fully replacing the hard palate. The orbital-maxillary cavity is obliterated with the latissimus dorsi and a scapular bony graft wrapped by latissimus muscle is used to reconstruct the inferior orbital rim and anterior maxillary wall (Fig. 2i,j). When the eyelids and/or adjacent skin are also resected, the chimeric latissimus dorsi is harvested as a myo-cutaneous component, in order to have the skin paddle replacing the cutaneous defect.

Dental rehabilitation of the scapular tip bone with osteo-integrated implants is controversial. Many reports claim the feasibility and reliability of placing dental implants in the scapular tip flap [23,24]. However, bone consistency and thickness are crucial to host dental implants and it is incontrovertible that the bone stock provided by the scapula is most frequently suboptimal, except in young and muscular males. Furthermore, in most patients, only the lateral scapular border might be suitable to host implants. In this light, thoughtful preoperative planning is crucial to optimize the correct in-setting for dental rehabilitation. The application of virtual surgical planning (VSP) has been recently described for several defects, including midface, mandible, and orbital floor [25–27]. VSP can be useful to plan the resection [28] and flap harvest osteotomies, and, in comparison to the free hand technique, is associated with better bone contouring, more precise flap in-setting, and is particularly helpful if dental rehabilitation is planned [25,29]. However, the need for postponing dental implants in case of planned adjuvant (chemo-)radiotherapy must be taken into account in such challenging cases.

For resections of the orbito-zygomatic complex, restoration of the anatomical contour and position of the malar eminence and orbital rim are critical [30]. In this scenario, the chimeric system of flaps based on the TDA allows for excellent restoration of shape and conformance. In fact, the bony component of the scapular tip provides a large flat bone that can be differently oriented with ‘greenstick’ fractures that are easily performed to obtain multiple orientations. The chimeric harvest is useful in cases in which soft tissues are required. Furthermore, the scapular bone can accept implant-retained orbital/nose prostheses after orbital exenteration and/or total rhinectomy [30].

## MANDIBULAR DEFECTS

The free fibula flap currently represents the first-choice reconstructive option in most instances of segmental mandibular resections. However, the SSSF, and especially the angular branch-based tip of scapula, offers a reliable alternative for patients who are not suitable for fibula harvesting due to previous limb trauma or severe vascular disease. Of note, the vascular pedicles nourishing the SSSF seems to be the least affected by atherosclerosis in the human body [31]. Moreover, other advantages of the scapular tip are reduced long-term donor site morbidity, early ambulation time, and, most of all, on the possibility of harvesting large and independent chimeric soft tissue components to address complex defects [32–34].

The mandible is a curved bone, and the lateral border of the tip is usually employed for linear mandible defects with complex soft-tissue requirements [32]; however, taking advantage of the similarity between the curvature of the scapular tip and the mandibular symphysis, this flap can be oriented horizontally to effectively reconstruct large anterior mandibular gaps with satisfactory results. In a recent series of anterior mandibular defects reconstructed with the scapular tip flap placed horizontally, cephalometric analyses revealed comparable measurements between preoperative and reconstructed mandibles [6^▪^].

The triangular shape of the scapular tip flap can be effectively used to reconstruct segmental resections of the mandibular angle, having the angle of the scapula to replace the angle of the mandible. A recent case series described the application of scapular tip free flap following mandibulectomy that included the condyle: the mandibular segment and scapular flap were cut using cutting guides and the scapular tip was placed into the glenoid fossa serving as new condyle, with good functional and aesthetic long-term outcomes [25].

## CONCLUSION

The SSSF represents a reliable option for a vast variety of head and neck oncologic defects, with no other donor site offering such a favorable toolbox of chimeric options. The relatively recent inclusion of the angular branch-based scapular tip flap as a source of vascularized bone has considerably widened the range of applications of this option for reconstructive purposes, thanks to the increased pedicle length with respect to the classic lateral border of the upper scapula based on the circumflex artery and subscapular vascular supply. The chimeric components of the SSSF benefit from considerable independency provided by the length of the named arteries arising from the main thoracodorsal pedicle. This offers a high degree of freedom to accomplish the desired flap in-setting according to a variety of possible orientation according to the specific needs of the case. The use of small denuded bony grafts nourished by surrounding vascularized muscles is a safe solution that well withstands postoperative adjuvant treatments, enhances facial contouring in maxillary reconstruction, and may also be efficient for orbital sustain to prevent postoperative diplopia. The possibility for simultaneous harvest during tumor ablation and a favorable postoperative donor site morbidity complete the convenience of this reconstructive solution, rendering the SSSF an essential tool for every head and neck surgical team dealing with complex oncologic defects.

## Acknowledgements

*None.*

### Financial support and sponsorship

*None.*

### Conflicts of interest

*There are no conflicts of interest.*

## REFERENCES AND RECOMMENDED READING

Papers of particular interest, published within the annual period of review, have been highlighted as:

▪ of special interest
▪▪ of outstanding interest
